# A Rare Case of Bullous Pemphigoid Secondary to Checkpoint Inhibitor Immunotherapy: A Tense Situation

**DOI:** 10.7759/cureus.16169

**Published:** 2021-07-04

**Authors:** Jacqueline T Wesolow, Samuel Jalali, Leah D Clark

**Affiliations:** 1 Internal Medicine, Moffitt Cancer Center, Tampa, USA; 2 Oncology, Moffitt Cancer Center, Tampa, USA

**Keywords:** bullous pemphigoid, immunotherapy, melanoma, immune checkpoint inhibitor, drug rash

## Abstract

Bullous pemphigoid is a serious and rare complication of immunotherapy. Here, we present a case of bullous pemphigoid secondary to ipilimumab/nivolumab checkpoint inhibitor therapy in a patient with metastatic melanoma. Immune checkpoint inhibitor therapy is more widely used now to treat cancer patients, bringing more challenging cases of adverse events associated with their use. Bullous pemphigoid can be a difficult diagnosis to make in the initial stages as the rash is similar to other red rashes before transforming into the typical appearance of a blister. As bullous pemphigoid can be a life-threatening adverse event, early identification is key to increasing patient survival.

## Introduction

Immune checkpoint inhibitors have emerged as a key player in effectively treating metastatic solid malignancies. These immunotherapies are a group of monoclonal antibodies that inhibit key proteins (e.g., programmed death-ligand 1, programmed cell death 1, and cytotoxic T-lymphocyte-associated protein 4) that allow tumor cells to avoid programmed cell death via antigen-specific T-cell immune responses, thereby allowing more targeted destruction of tumor cells [1,2]. However, this increase in T-cell activation also leads to a release of inflammatory cytokines causing multiple immune-related adverse events (irAEs) including, but not limited to, colitis, dermatitis, hepatitis, hypophysitis, and pneumonitis [3]. irAEs are usually graded by their severity, which dictates treatment, ranging from holding immunotherapy with close monitoring to starting high-dose steroids [4,5]. Dermatologic toxicities are the most common, presenting in 50% of patients on immunotherapy, and present the earliest (approximately three to six weeks from the initial dose). Most common presentations include maculopapular rashes, pruritis, lichenoid eruptions, and eczema. Bullous pemphigoid, an autoimmune disorder caused by autoantibodies against hemidesmosomes that maintain the dermal-epidermal junction, is a rare and more severe dermatologic manifestation associated with immunotherapy resulting in tense bullae [4,5].

We present a rare case of severe bullous pemphigoid in a metastatic melanoma patient that was secondary to ipilimumab/nivolumab checkpoint inhibitor therapy, requiring high-dose intravenous steroids and rituximab.

## Case presentation

A 63-year-old male was admitted to our institution for progressively worsening skin blisters on his trunk, hands, and feet. He was diagnosed with mucosal melanoma of the hard palate in 2009. He never smoked cigarettes and denied the use of recreational drugs or alcohol. He had a wide excision of the hard palate followed by radiation therapy in 2009, which was followed by right neck dissection and adjuvant radiation therapy in 2010. He was initially in remission but was found to have recurrent metastatic melanoma involving the lungs, mediastinum, and spleen (confirmed on CT-guided biopsy of the left lung). Between 2019 and 2020, the patient underwent four cycles of ipilimumab/nivolumab, two cycles of nivolumab monotherapy, a clinical trial consisting of nivolumab plus avadomide, and a second clinical trial with ceritinib/trametinib. In early 2021, the patient was rechallenged with ipilimumab/nivolumab due to the progression of the disease. The first dose was given approximately 17 days prior to the initial presentation (i.e., six weeks prior to inpatient admission).

**Figure 1:**
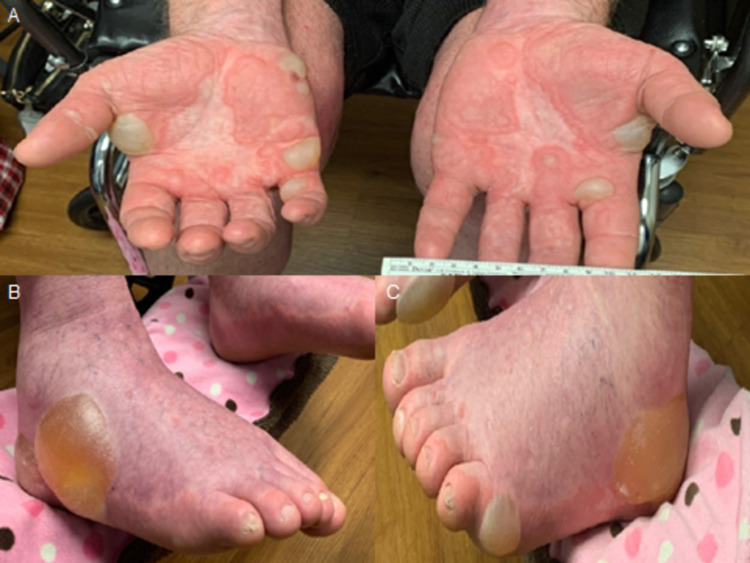
(A) Multiple tense bullae on both hands with surrounding erythema. (B) Tense bullae along the lateral surface of the right foot containing sanguineous fluid. (C) Tense bullae along the lateral surface of the left foot containing sanguineous fluid.

**Figure 2:**
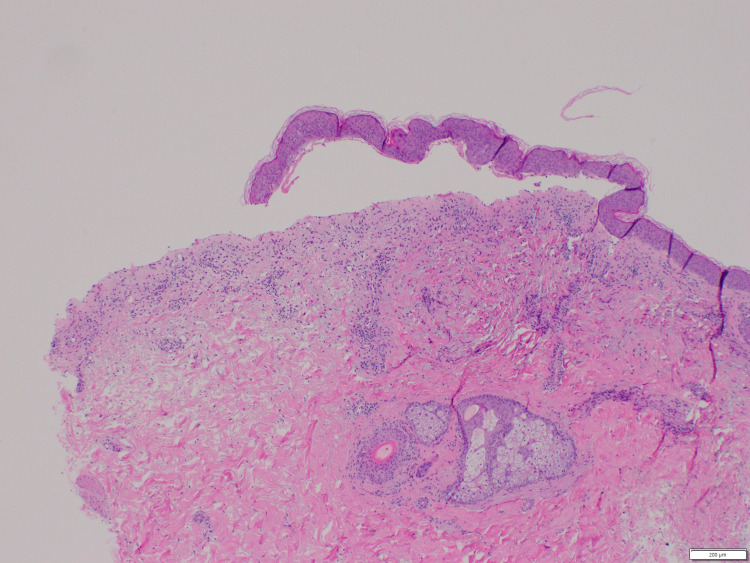
Subepidermal and suprabasilar vesiculation with underlying dermal inflammatory infiltrate with numerous eosinophils.

He initially presented to his oncologist four weeks prior to admission with a diffuse maculopapular rash that progressed to tense bullae, most significantly in the palmar and plantar surfaces of the hands and feet, respectively (Figure 1). Lesions ranged from 1 to 5 cm, contained clear serous fluid, were painful and pruritic, and limited the patient from being able to stand, ambulate, or perform any of his instrumental activities of daily living. Scattered smaller bullae on the abdomen and thighs were also noted. Due to concern for immune checkpoint inhibitor-related bullous dermatitis, the patient’s second cycle of ipilimumab/nivolumab was held. He was initially treated with a methylprednisolone dose pack and topical clobetasol but showed no improvement. Dermatology was consulted and performed a punch biopsy on a right abdominal lesion, which revealed subepidermal and suprabasilar vesiculation with underlying dermal inflammatory infiltrate with numerous eosinophils, with direct immunofluorescence studies consistent with bullous pemphigoid (IgG4 and C3 deposition along the linear basement membrane zone) (Figure 2).

Serum enzyme-linked immunosorbent assay showed an elevated serum IgG BP 180 antibody level of 167 units (elevated if >8 units), while IgG BP 230 levels were within normal limits. Due to the progression of his symptoms, the patient was admitted to our inpatient service and started on oral doxycycline, intravenous (IV) methylprednisolone 1 mg/kg/day, and a 1,000 mg IV rituximab infusion. On admission, the patient’s vital signs were normal and he was afebrile. Labs were unremarkable, except for an elevated white blood cell count of 10,280/uL. Over the five-day hospital course, the patient’s skin bullae decreased in size. Pain improved with applying cold compresses to his hands and feet. Dermatology offered to perform therapeutic sterile rupture of bullae, but the patient declined. The patient was discharged on a prednisone taper, started on famotidine for pectic ulcer prophylaxis, trimethoprim-sulfamethoxazole for *Pneumocystis jirovecii* prophylaxis, and a one-month course of doxycycline, with tentative plans to receive a second infusion of rituximab two weeks after the prior dose.

## Discussion

Surgery with wide excision is typically curative in patients with early melanoma. These patients usually do not require systemic therapy. In patients where melanoma has reached the lymph nodes, adjuvant treatment with an immune checkpoint inhibitor may be indicated. Our patient with stage IV melanoma was treated with a combination of ipilimumab and nivolumab checkpoint inhibitor therapy. In 2011, ipilimumab was the first FDA-approved immunotherapy drug for use in metastatic melanoma [6]. Nivolumab followed shortly for use in metastatic melanoma as well [6]. Although the development of these drugs has transformed cancer care with increased patient survival rates, it has also brought about various side effects. As more patients receive these immunotherapy drugs, the more adverse, at times life-threatening, side effects become more common. Immunotherapy-related side effects include a spectrum of cutaneous, neurologic, hepatic, and cardiac events [5]. Our patient suffered a painful, blistering skin reaction to a combination of ipilimumab and nivolumab called bullous pemphigoid.

Bullous pemphigoid is a rare blistering skin disease. Pemphigoid blisters are tense fluid-filled sacs [4]. These sacs can contain either clear or bloody fluid [4]. The wall of the blister is usually firm and thin. Pemphigoid blisters can rupture or become infected causing them to change their appearance to that of an ulcer. Bullous pemphigoid blisters typically form in the subepidermal layer of the skin [4]. Before becoming blisters, they may present as a pruritic red rash [4]. They can either rapidly transform into blisters or progressively change over a period of weeks to months. If a patient on immunotherapy presents with a rash that is not improving with topical steroids, one should suspect bullous pemphigoid. In these cases, it is recommended that a skin biopsy is obtained. A perilesional biopsy is recommended within 1 cm from the bulla [7]. The biopsy should be obtained from the surrounding nonbullous part of the lesion [7].

Pemphigoid blisters are generally in the flexor regions of the body such as the axilla, but they can form anywhere on the body including the mucosa of the lips [4]. Patients can present with only a few or multiple widespread pemphigoid blisters. They can present as a red rash before transforming into a blister. As a red rash is a common presentation of many skin diseases, one should be aware of this rare condition. Other known cutaneous side effects of immunotherapy include lichenoid eruptions, Stevens-Johnson syndrome, erythema multiforme, vitiligo skin hyperpigmentation, and psoriasiform rash [8]. The number of cases of Stevens-Johnson syndrome secondary to immunotherapy use is similar to bullous pemphigoid [9].

According to the National Comprehensive Cancer Network guidelines, treatment is determined by grading the severity of disease from grade 1 to the most severe which is grade 4 [10]. Each grade is based on the percentage of the total body surface area (BSA) affected. In grade 1, blisters cover <10% BSA, in grade 2, blisters cover 10-30% BSA, and in grade 3 they cover >30% BSA (Table 1). Management for all grades includes holding immunotherapy. However, for grades 2-3, it is recommended that immunotherapy is discontinued permanently. Grade 1 is treated with high-potency topical steroids, whereas grades 2-4 require IV steroid therapy. Rituximab, as given in our patient, is recommended in patients not responding to IV steroids after three days. All grades require dermatology consultation.

**Table 1:** Grading of bullous pemphigoid based on the total BSA affected. BSA: body surface area

Grade	BSA
1	<10%
2	10–30 %
3 and 4	>30%

Rituximab is an anti-CD20 monoclonal antibody [11]. Rituximab therapy is typically given if patients are not responding to IV steroids after three days. One retrospective study on a small group of 20 patients treated with rituximab showed that 15 patients went into remission. It was found in this and other studies that patients have a high rate of remission in cases treated with rituximab [11].

## Conclusions

With the increasing use of immune checkpoint inhibitors in treating metastatic malignancies, clinicians should be made aware of potential irAEs, especially dermatologic manifestations. Bullous pemphigoid is a rare autoimmune skin blistering disease that can occur as a result of immunotherapy. It can have deleterious effects on a patient’s quality of life. Therefore, prompt discontinuation of immunotherapy and coordination with oncology and dermatology are essential to treatment, especially in severe cases refractory to steroids.
